# FLOURY ENDOSPERM 2 Coordinates Starch Biosynthesis to Maintain Endosperm Structural Integrity in Rice

**DOI:** 10.3390/genes17010063

**Published:** 2026-01-05

**Authors:** Hye-Mi Lee, Jin-Young Kim, Hak-Dong Kim, Hak-Soo Kim, Jong-Geun Park, Yu-Jin Jung, Kwon-Kyoo Kang

**Affiliations:** 1 Division of Horticultural Biotechnology, Hankyong National University, Anseong 17579, Republic of Korea; 2 Institute of Genetic Engineering, Hankyong National University, Anseong 17579, Republic of Korea

**Keywords:** *FLOURY ENDOSPERM 2* (*FLO2*), rice endosperm, starch physicochemical properties, amylopectin chain-length distribution, flour pasting behavior, CRISPR/Cas9

## Abstract

Background/Objectives: FLOURY ENDOSPERM 2 (FLO2) is known to affect rice endosperm development and starch quality, yet its role in determining flour physicochemical behavior and endosperm structural integrity has not been quantitatively defined. This study aimed to elucidate how loss of FLO2 function alters starch organization and functional properties of rice flour. Methods: Two independent homozygous, T-DNA-free *OsFLO2* knockout lines were generated in the japonica cultivar Dongjin using CRISPR/Cas9. Grain appearance was evaluated in mature seeds. Flour physicochemical properties were analyzed by Rapid Visco Analyzer (RVA) and differential scanning calorimetry (DSC). Amylopectin chain-length distribution was determined by isoamylase debranching followed by HPAEC-PAD, and endosperm microstructure was examined by scanning electron microscopy. Results: *OsFLO2* mutants exhibited floury, opaque endosperms, with chalkiness increasing from 4.8% in the WT to 27–29%. RVA analysis showed a marked reduction in peak viscosity (1193 cP to 263–293 cP) and a decrease in pasting temperature (77.2 °C to 68.9–70.5 °C). DSC indicated a tendency toward reduced gelatinization enthalpy in the mutants. These changes were associated with a reduced proportion of short amylopectin chains (DP 6–12), decreased long chains (DP ≥ 37), and a relative increase in intermediate-long chains (DP 25–36), along with disrupted granule packing and a 1.33–1.36-fold increase in endosperm porosity. Conclusions: These results demonstrate that FLO2 plays an important role in maintaining the structural integrity of rice endosperm by harmonizing the microstructure of amylopectin with the thermal and gelatinization properties of starch.

## 1. Introduction

Rice endosperm quality is a major determinant of grain appearance, processing performance, and eating quality, and is primarily governed by the structural organization of starch accumulated during endosperm development [1,2,3,4]. As the predominant storage carbohydrate, starch constitutes most of the endosperm dry weight, and variations in its molecular architecture and granule packing directly influence chalkiness and functional behavior of rice flour [2,3,4]. Chalky endosperms are characterized by loosely packed starch granules and enlarged intergranular air spaces, which reduce endosperm density and impair grain quality [2,4]. At the molecular level, these structural features arise from how starch biosynthesis is coordinated during endosperm development. Endosperm starch structure is not dictated by the activity of a single enzyme but rather emerges from the balanced coordination of multiple starch-biosynthetic enzymes. In cereal endosperm, starch biosynthesis is mediated by the concerted action of granule-bound starch synthase I (GBSSI), soluble starch synthases (SSI, SSIIa and SSIIIa), starch branching enzymes (BEI and BEIIb), and debranching enzymes such as isoamylase (ISA1) [1,5,6,7,8,9]. The relative activities and coordination among these enzymes determine amylopectin chain-length (degree of polymerization, DP) distribution, which in turn underlies starch granule organization and endosperm structure [8,9,10]. Genetic disruption of key enzymes, particularly SSIIa and BEIIb, shifts amylopectin DP profiles toward increased short chains and reduced long chains, structural changes that are frequently accompanied by altered starch gelatinization behavior and flour pasting characteristics [10,11]. Collectively, these studies suggest that amylopectin chain-length distribution represents an important molecular feature linking starch biosynthetic regulation to endosperm structure and grain quality traits. Previous studies have established that amylopectin chain-length distribution is a key determinant of starch granule organization and endosperm structure, arising from the coordinated activities of multiple starch-biosynthetic enzymes [12,13]. These observations suggest that starch organization in the endosperm cannot be fully explained by the activities of individual biosynthetic enzymes alone. This limitation implies that additional non-catalytic components may be involved in coordinating starch biosynthesis during endosperm development. In this context, *OsFLO2* encodes a tetratricopeptide repeat (TPR)–containing protein that was identified through map-based cloning of rice mutants displaying floury and opaque endosperms [14]. Unlike core starch-biosynthetic enzymes, FLO2 lacks known catalytic domains, suggesting that it does not directly participate in starch synthesis [14]. The classical *flo2* mutant exhibits reduced grain weight, abnormal starch accumulation, and a chalky grain appearance, indicating that disruption of FLO2 consistently affects starch-related endosperm phenotypes [14]. Subsequent allelic analyses further showed that mutations in *FLO2* alter amylose content and grain quality [15]. Consistent with these observations, CRISPR/Cas9-mediated mutagenesis studies confirmed that targeted disruption of *FLO2* reproducibly induces floury endosperm phenotypes [16]. Despite consistent phenotypic outcomes across independent *flo2* alleles, how FLO2 influences starch organization at the molecular and structural levels remains unclear. Previous studies have primarily focused on compositional parameters or external grain traits, whereas the consequences of FLO2 perturbation for amylopectin DP distribution and starch granule organization have not been systematically examined. Given that starch granule structure emerges from coordinated enzyme activities rather than isolated compositional changes, examining these features provides a more direct window into FLO2 function during endosperm development.

In this study, we generated independent *OsFLO2* loss-of-function lines in the japonica rice cultivar Dongjin using CRISPR/Cas9-mediated genome editing and examined the effects of FLO2 disruption on starch organization. By combining amylopectin chain-length analysis with assessments of starch functional behavior and granule morphology, we sought to clarify how FLO2 contributes to the coordination of starch biosynthesis and the maintenance of endosperm structural integrity. This study places FLO2 function within a structural framework by examining how its disruption affects starch organization and grain quality.

## 2. Materials and Methods

### 2.1. Plant Materials and Growth Conditions

Dongjin (*Oryza sativa* L. ssp. *japonica*) was used as the wild-type (WT) control and as the genetic background for generating the *OsFLO2* genome-edited lines. Plants, including regenerated genome-edited individuals, were cultivated in contained greenhouse facilities and experimental rice fields at Hankyong National University (Anseong, Republic of Korea), as previously described [17]. For developmental analyses, developing caryopses were collected at defined time points after flowering (0, 5, 10, 15, 20, and 25 days after flowering, DAF). Harvested tissues were immediately frozen in liquid nitrogen and stored at −80 °C until use. Mature seeds were harvested at full maturity, air-dried, and further dried to approximately 14% moisture content before storage under dry conditions at 4 °C prior to downstream analyses [18].

### 2.2. CRISPR/Cas9 Vector Construction and Rice Transformation

The genomic DNA sequence of *OsFLO2* was obtained from the NCBI and Gramene databases. One CRISPR target site was selected in exon 5 and another in exon 11 of *OsFLO2* using the CRISPR RGEN Tools platform (http://www.rgenome.net/ accessed on 12 March 2024) [19] (Supplementary Table S1). Each sgRNA was designed adjacent to a canonical 5′-NGG-3′ PAM motif, and oligonucleotides corresponding to the two sgRNAs were synthesized by Bioneer Co., Ltd. (Daejeon, Republic of Korea). The annealed sgRNA oligonucleotide pairs were cloned into the pBOsC binary vector through AarI restriction enzyme digestion to generate the CRISPR/Cas9 expression construct [20]. The recombinant plasmid was introduced into *Agrobacterium tumefaciens* strain EHA105, and rice embryogenic calli were transformed using an *Agrobacterium*-mediated infection and regeneration procedure [21]. Infected calli were selected on 2N6 medium supplemented with hygromycin and carbenicillin, and resistant callus lines were maintained through multiple subculture cycles (Supplementary Figure S1). Regenerated T_0_ plants were transferred to soil in the greenhouse and grown to maturity for subsequent genotyping and phenotypic analyses.

### 2.3. Genotyping and Mutation Validation

Genomic DNA was extracted from approximately 100 mg of young leaf tissue using the DNA Quick Plant Kit (Inclone, Jeonju, Republic of Korea), following the manufacturer’s protocol. The genomic regions encompassing the CRISPR target sites in exon 5 and exon 11 of *OsFLO2* were amplified by PCR using gene-specific primers. For mutation analysis, PCR amplicons were submitted to Bioneer Co., Ltd. (Daejeon, Republic of Korea) for next-generation sequencing (NGS) (Supplementary Figure S2, Supplementary Table S3). Sequencing reads were analyzed using the Cas-Analyzer tool (http://www.rgenome.net/cas-analyzer/ accessed on 21 June 2024) to determine indel types, editing efficiency, and zygosity [22,23]. T-DNA–free lines were identified by PCR using bar gene–specific primers (Supplementary Figure S2, Supplementary Table S3). Plants lacking amplification of the *bar* gene were selected as transgene-free and used for subsequent phenotypic and molecular analyses.

### 2.4. Grain Trait Measurement

Mature grains were harvested at DAF58, air-dried, and further dehydrated in a 35 °C oven for 3 days prior to analysis. Chalkiness percentage and starch granule size were quantified from cross-sectional and SEM images using ImageJ software (version 1.54g) following previously described procedures. One thousand-grain weight was measured using fully matured and dehulled seeds after moisture equilibration, and the filled grain ratio was determined by counting filled and unfilled spikelet from at least three independent panicles. For endosperm cross-sectional imaging, grains were hand-cut using a sterile medical scalpel, briefly pre-dried to stabilize the cutting surface, and examined under a stereomicroscope (SMZ800N, Nikon, Tokyo, Japan).

### 2.5. Preparation of Rice Endosperm Flour

Mature rice grains were harvested at full maturity and air-dried prior to analysis. Dehulled seeds were milled into fine flour using a laboratory grinder, and the resulting rice flour was passed through a 100-mesh sieve to obtain uniformly sized particles. The flour samples, which contain native starch embedded within the endosperm matrix, were stored in airtight containers at room temperature until use. Unless otherwise stated, all subsequent analyses were performed using rice flour without chemical starch isolation or protein removal, as commonly applied in rice grain quality and starch property studies [24].

### 2.6. Rapid Visco Analyzer (RVA) Analysis

Pasting properties of starch were analyzed using a RVA 4500 (Perten Instruments, Hägersten, Sweden) at the Bio-Polymer Advanced Materials Core-Facility Center, Sejong University. 3.0 g of rice flour (12% moisture basis) with 25 mL of distilled water in an RVA canister. The measurement was conducted using a standard heating–cooling cycle programmed as follows: holding at 50 °C for 1 min, heating to 95 °C, followed by cooling back to 50 °C at a constant rate. A holding time of 150 s was applied at the maximum temperature. Stirring speed was set to 960 rpm for the first 10 s and then maintained at 160 rpm throughout the remainder of the run. Viscosity parameters—peak viscosity, trough viscosity, breakdown, final viscosity, setback, and pasting temperature—were recorded automatically using the RVA software (version 3.0). All analyses were performed using three independent biological replicates.

### 2.7. Differential Scanning Calorimetry (DSC)

Thermal properties of starch were analyzed by DSC at the Core Research Facility of Hankyong National University using a DE/DSC204F1 calorimeter (NETZSCH, Selb, Germany). Rice flour samples (5–10 mg) were weighed into standard aluminum pans and mixed with distilled water at a 1:2 (*w*/*w*) starch-to-water ratio before sealing. The pans were equilibrated for 1 h at room temperature and subjected to a heating–cooling program from 20 °C to 250 °C and back to 20 °C at a constant scan rate of 20 °C/min. An empty sealed pan served as the reference. The onset (To), peak (Tp), and conclusion (Tc) temperatures were recorded from the endothermic transition, and gelatinization enthalpy (ΔH) was calculated by integrating the area under the endothermic peak using the instrument software. All measurements were performed in triplicate using independent biological samples.

### 2.8. HPAEC-PAD Analysis of Amylopectin Chain-Length Distribution

Amylopectin chain-length distribution was determined using HPAEC-PAD. Purified starch was first defatted by consecutive washes with 85% (*v*/*v*) methanol and absolute ethanol, air-dried, and subsequently gelatinized by incubation in 0.25 M NaOH for 30 min. The solution was neutralized with 1 M HCl and debranched with isoamylase from *Pseudomonas amyloderamosa* (Megazyme, Bray, Ireland) at 40 °C for 12 h in 50 mM sodium acetate buffer (pH 4.0). After enzyme inactivation at 95 °C for 10 min, samples were filtered through a 0.22 µm membrane and subjected to chromatographic analysis. Debranched glucans were separated on a CarboPac PA-100 analytical column (4 × 250 mm) equipped with a PA-100 guard column (4 × 50 mm) using a Dionex ICS system with pulsed amperometric detection. Elution was performed with a binary mobile phase consisting of 150 mM NaOH (solvent A) and 150 mM NaOH containing 500 mM sodium acetate (solvent B) under a linear gradient from 0 to 50% solvent B over 65 min at a flow rate of 1.0 mL/min. Relative molar percentages were calculated for each chain-length class, and three independent biological replicates were analyzed per genotype.

### 2.9. Scanning Electron Microscopy (SEM)

For microstructural analysis, fractured grain surfaces and isolated starch granules were prepared following previously established sample-preparation procedures [25,26] and submitted to the Core Research Facility of Hankyong National University for scanning electron microscopy (SU3800, Hitachi, Tokyo, Japan). Samples were sputter-coated with platinum (Pt) under high-vacuum conditions, and images were acquired using a FE-SEM operated at 3 kV with an SE detector at ×5000 and ×10,000 magnifications. Representative images from three biological replicates were collected for morphological assessment.

### 2.10. Quantitative RT-PCR Analysis

Total RNA was extracted from developing rice endosperm using the FavorPrep™ Plant Total RNA Mini Kit (FAPRK 001-1, Favorgen, Ping-Tung, Taiwan) according to the manufacturer’s instructions. RNA integrity and concentration were assessed by agarose gel electrophoresis and spectrophotometric analysis. A total of 1 µg RNA was reverse transcribed into cDNA using the RevertAid First Strand cDNA Synthesis Kit (Thermo Fisher Scientific, Waltham, MA, USA). Quantitative PCR was performed on a CFX96 Real-Time PCR System (Bio-Rad, Hercules, CA, USA) using the AccuPower^®^ 2× GreenStar™ qPCR Master Mix (Bioneer, Daejeon, Republic of Korea). *OsACTIN* was used as an internal reference gene, as it has been previously reported to show stable expression in rice qRT-PCR analyses [27]. Gene expression levels were normalized to *OsACTIN*, and relative expression was calculated using the 2^−ΔΔCt^ method [28], with WT samples serving as the reference. Expression profiling targeted starch-biosynthesis–related genes, including granule-bound starch synthases (*GBSSI* and *GBSSII*), soluble starch synthases (*SSIIa*, *SSIIIa* and *SSIVa*), branching enzymes (*BEI*, *BEIIa* and *BEIIb*), and debranching enzymes (*ISA* and *PUL*) (Supplementary Table S3). Primer specificity was confirmed through melt-curve analysis, and all measurements were conducted using three independent biological replicates, each analyzed in technical triplicate.

### 2.11. Multivariate Statistical Analysis

Multivariate statistical analysis was performed to examine global patterns and co-variation among physicochemical traits and gene expression data. Principal component analysis (PCA) was conducted using RStudio (R version 4.5.2). All variables were auto-scaled (mean-centered and scaled to unit variance) prior to PCA to account for differences in measurement units and variance magnitudes. PCA score and loading plots were used to visualize genotype-dependent clustering patterns and to identify major trait contributors to endosperm quality variation. Analyses were conducted using averaged values from three independent biological replicates.

### 2.12. Statistical Analysis

All statistical analyses were performed using RStudio (R version 4.5.2) unless otherwise noted. For phenotypic, physicochemical, and gene-expression measurements, biological replicates were defined as samples obtained from independent plants, whereas technical replicates referred to repeated measurements of the same biological sample within an assay. Differences between WT and *OsFLO2* mutants were evaluated using Student’s *t*-test for pairwise comparisons or one-way ANOVA followed by Tukey’s post hoc test when more than two groups were compared. A significance threshold of *p*-value was applied unless otherwise specified. Statistical significance was denoted as follows: *, *p* < 0.05; **, *p* < 0.01; and ***, *p* < 0.001. Data are presented as the mean ± standard deviation (SD), and error bars in all figures represent SD derived from biological replicates.

## 3. Results

### 3.1. Generation of OsFLO2 Knockout Lines

**Figure 1:**
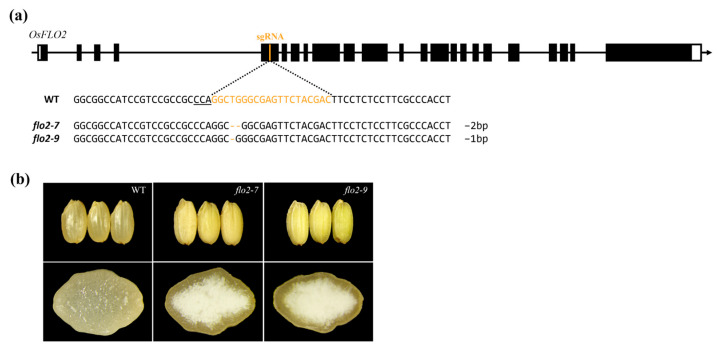
CRISPR/Cas9-mediated knockout of *OsFLO2* and grain phenotypes of rice mutants. (**a**) Schematic representation of the *OsFLO2* gene structure. Exons (black boxes), introns (lines), and the Cas9 sgRNA target site located within exon 5. The aligned WT and mutant sequences indicate the edited region, including the PAM motif and the indel types identified in *flo2-7* and *flo2-9*. PAM sequence indicated on the line below. (**b**) Representative grain morphology of the WT and *OsFLO2* mutants. Compared with WT, both *flo2-7* and *flo2-9* exhibit opaque, chalky endosperms, as shown in whole-grain images (upper panel) and transverse grain sections (lower panel).

CRISPR/Cas9-mediated genome editing targeting exon 5 of *OsFLO2* was used to generate transgenic rice plants. In the T_0_ generation, edited individuals were initially screened by PCR amplification of T-DNA–derived regions to identify genome-edited plants (Figure 1a, Supplementary Figure S1 and Supplementary Table S2). Deep sequencing analysis was then performed to determine the mutation types present in individual T_0_ plants, and homozygous edited lines were selected and designated *flo2-7* and *flo2-9* (Supplementary Figure S2A, Supplementary Table S2). Following self-pollination, the T_1_ generation was examined to confirm the absence of residual T-DNA, and T-DNA–free individuals were identified (Supplementary Figure S2B). As a result, two homozygous, T-DNA–free knockout lines were established, harboring a 2 bp deletion (*flo2-7*) and a 1 bp deletion (*flo2-9*), respectively (Supplementary Figure S2). Both deletions disrupt the coding frame of *OsFLO2* and generate frameshift mutations.

### 3.2. Grain Phenotypes of OsFLO2 Knockout Lines

Both *flo2-7* and *flo2-9* carry small deletions within exon 5 of *OsFLO2* that disrupt the coding frame and result in frameshift mutations. Despite the difference in deletion size (2 bp in *flo2-7* and 1 bp in *flo2-9*), the two mutant lines exhibited highly comparable grain phenotypes. Both *flo2-7* and *flo2-9* produced opaque, floury endosperms and showed a pronounced increase in chalkiness compared with the WT, with chalkiness ratios of 27.3% and 29.1%, respectively, whereas WT grains exhibited a chalkiness of 4.8% (Table 1). In addition, both mutants displayed modest but consistent reductions in 1000-grain weight relative to WT, while the filled grain ratio was only slightly affected. The close phenotypic similarity observed between *flo2-7* and *flo2-9*, despite their independent mutation events, indicates that these endosperm defects are attributable to disruption of *OsFLO2* rather than to allele-specific or background-dependent effects. Accordingly, both knockout lines were used in parallel for subsequent analyses to assess reproducible effects of *OsFLO2* loss on starch organization and endosperm properties.

### 3.3. Altered Pasting and Thermal Properties

**Figure 2:**
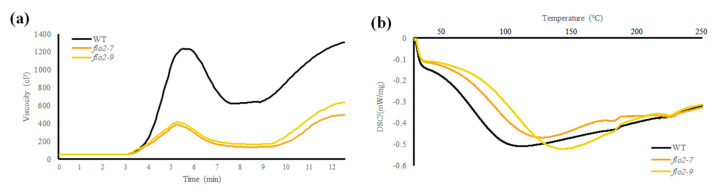
Loss of *OsFLO2* reduces pasting viscosity and alters thermal gelatinization properties. (**a**) RVA pasting profiles of WT and *flo2* mutant lines, showing the time-dependent changes in paste viscosity during heating and cooling cycles. (**b**) DSC thermograms of WT and *flo2* mutants, illustrating their gelatinization endotherms across the temperature scan. Data represent the mean (n = 3).

RVA analysis revealed clear alterations in the pasting behavior of *OsFLO2* mutant lines compared with the WT. Both *flo2-7* and *flo2-9* exhibited markedly reduced peak viscosity, trough viscosity, and final viscosity, accompanied by lower breakdown values and decreased pasting temperatures (Figure 2a; Table 2). Overall, the RVA profiles of the mutant lines showed attenuated viscosity development throughout the heating–cooling cycle, indicating weakened pasting performance. Consistent with these changes, DSC analysis showed a reduction in gelatinization enthalpy (ΔH) in the *flo2* mutants relative to WT, together with a modest decrease in onset temperature (To) (Figure 2b; Table 2). These results indicate that disruption of *OsFLO2* affects the thermal behavior of endosperm starch, in line with the altered pasting properties observed by RVA.

### 3.4. Changes in Amylopectin Chain Length Distribution

**Figure 3:**
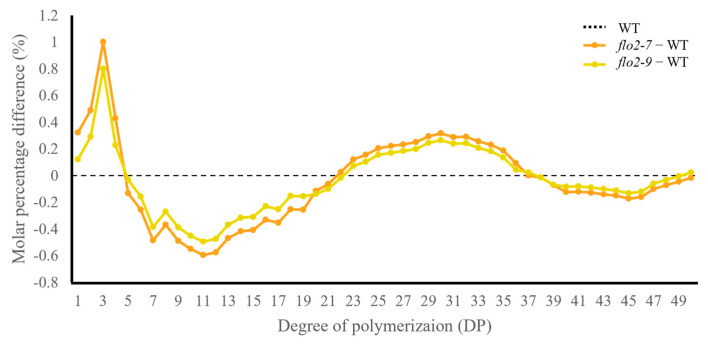
*OsFLO2* disruption alters amylopectin chain–length distribution in rice endosperm. The molar percentage differences in amylopectin glucan chains with DP 1–50 were plotted for *OsFLO2* mutants relative to the WT. The WT baseline is shown as a dotted line. Data represent the mean of three biological replicates (n = 3).

HPAEC–PAD analysis revealed distinct alterations in amylopectin chain-length distribution in *OsFLO2* mutant lines compared with the WT. Both *flo2-7* and *flo2-9* showed a reduced proportion of short glucan chains (DP 6–12), together with decreases in intermediate chains (DP 13–24) and long chains (DP ≥ 37) (Figure 3). In contrast, glucan chains within the intermediate-long range (DP 25–36) were relatively increased in the mutant lines. Across the defined DP classes, the amylopectin profiles of *flo2* mutants consistently deviated from those of WT, indicating a redistribution of chain-length composition toward intermediate-long chains rather than a uniform change across all DP ranges. These trends were reproducibly observed in independent biological replicates.

### 3.5. Microstructural Alterations Observed by SEM

**Figure 4:**
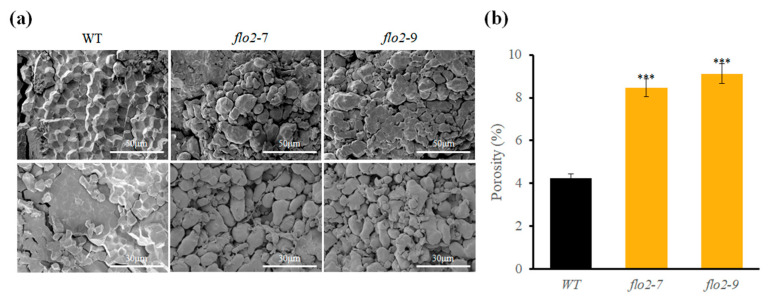
SEM reveals altered granule morphology and increased porosity in *OsFLO2* mutants. (**a**) SEM images of endosperm cross-sections and purified starch granules from WT, *flo2-7*, and *flo2-9*. Images were captured at 5000× (upper row) and 10,000× (lower row) magnification. (**b**) Porosity (%) quantified from SEM micrographs using image segmentation–based analysis. Bars represent the SD from three biological replicates (n = 3). Statistical significance was determined using Student’s *t*-test (***, *p* < 0.001).

Scanning electron microscopy revealed clear differences in starch granule morphology between the WT and *OsFLO2* mutants. WT endosperm contained tightly packed, polygonal granules with smooth surfaces, whereas granules from *flo2-7* and *flo2-9* exhibited irregular contours, surface fissures, and visibly enlarged intergranular spaces (Figure 4). SEM observations of purified starch granules further supported these differences, showing disrupted granule packing and more pronounced surface roughness in the mutant lines. Quantitative measures confirmed a significant increase in porosity index in both mutants, with values approximately 35% higher than those of WT (Table 3).

### 3.6. Transcriptional Reprogramming of Starch-Related Genes

**Figure 5:**
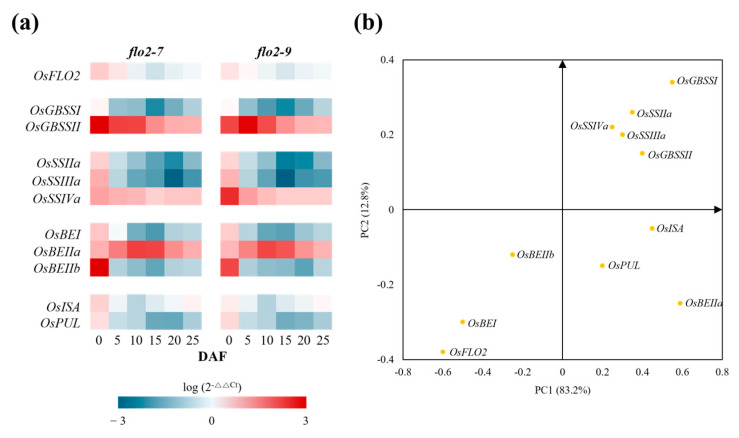
Transcriptional changes in starch biosynthetic genes in response to *OsFLO2* loss. (**a**) Heatmap showing the temporal expression profiles of starch biosynthetic genes in *flo2-7* and *flo2-9* across DAF. Gene expression values were calculated as log_10_(2^−ΔΔCt^) relative to WT, with red and blue gradients indicating higher and lower transcript abundance, respectively. (**b**) PCA regression biplot displaying the distribution of starch biosynthetic genes along the first two principal components (PC1 and PC2), which together explain the majority of the variance. Gene vectors and sample projections are plotted to visualize multivariate relationships between transcript abundance and RVA parameters. Data were derived from three biological replicates (n = 3).

qRT-PCR analysis revealed broad transcriptional alterations in starch biosynthetic pathways in the *OsFLO2* mutants. *GBSSI*, *SSIIa*, *SSIIIa*, *BEI*, *BEIIb*, *PUL*, and *ISA* displayed reduced transcript abundance across multiple developmental stages, whereas *GBSSII*, *SSIVa*, and *BEIIa* were consistently upregulated in both *flo2-7* and *flo2-9* (Figure 5a). To further examine the relationships between gene expression and starch functional properties, PCA was conducted to evaluate multivariate associations between transcriptional profiles and RVA-derived pasting parameters. The two primary components represented the major axes of variation, with PC1 reflecting overall differences in pasting behavior and PC2 capturing secondary variation independent of PC1 (Figure 5b). Genes involved in amylose synthesis (*GBSSI* and *GBSSII*) and the elongation of intermediate-to-long amylopectin chains (*SSIIa*, *SSIIIa* and *SSIVa*) were positioned toward the positive direction of PC1, whereas genes contributing to the formation of shorter glucan branches (*BEIIb*) mapped toward the negative PC1 axis. PC2 further distinguished genes associated with branch trimming and granule remodeling, including *ISA*, *PUL*, and *BEIIa*. Collectively, the PCA loadings indicate that coordinated transcriptional shifts among starch metabolic genes correspond to the variation in RVA-derived pasting properties.

## 4. Discussion

In this study, we investigated the role of *OsFLO2* in rice endosperm development by integrating phenotypic, physicochemical, structural, and transcriptional analyses of two independent CRISPR/Cas9-generated knockout lines. Both *flo2-7* and *flo2-9* carried small deletions within exon 5 that differed in size but resulted in comparable loss-of-function mutations (Figure 1a; Supplementary Figure S2). Despite their independent mutational origins, the two lines exhibited nearly identical grain phenotypes, including opaque, floury endosperms and a marked increase in chalkiness relative to the WT (Figure 1b and Table 1). The close agreement between these alleles supports the conclusion that the observed defects are attributable to disruption of *OsFLO2* itself rather than allele-specific or background-dependent effects, consistent with previous reports on classical *flo2* mutants and natural or genome-edited alleles [14,15,29,30]. The pronounced increase in chalkiness observed in both *OsFLO2* mutants was accompanied by substantial changes in starch functional properties. RVA analysis revealed strongly reduced peak, trough, and final viscosities, together with lower breakdown values and decreased pasting temperatures (Figure 2a; Table 2). These attenuated pasting profiles indicate weakened viscosity development and reduced paste stability, features commonly associated with chalky rice endosperm and impaired starch granule organization [24,25]. In parallel, DSC analysis showed reduced gelatinization enthalpy and a modest shift in onset temperature in the mutant lines (Figure 2b; Table 2), suggesting altered thermal stability of endosperm starch. The consistency of these functional changes across two independent knockout lines further indicates that *OsFLO2* disruption reproducibly affects starch behavior at the bulk flour level. At the molecular level, *OsFLO2* loss resulted in a distinct redistribution of amylopectin chain-length classes rather than a uniform directional shift. Both *flo2-7* and *flo2-9* exhibited reduced proportions of short (DP 6–12), intermediate (DP 13–24), and long chains (DP ≥ 37), accompanied by a relative enrichment of intermediate-long chains (DP 25–36) (Figure 3). This pattern differs from the stereotypical amylopectin DP changes observed in mutants of core starch-biosynthetic enzymes such as SSIIa or BEIIb [31], suggesting that *OsFLO2* does not directly control a specific elongation or branching step. Instead, the altered chain-length distribution likely reflects indirect perturbation of the coordinated activities that shape amylopectin architecture during endosperm development. Structural analysis by SEM provided a physical basis for the altered starch properties and grain appearance observed in *OsFLO2* mutants. Compared with the densely packed, polygonal starch granules of WT endosperm, *flo2-7* and *flo2-9* displayed irregular granule surfaces, fissures, and enlarged intergranular spaces (Figure 4a). Quantitative image analysis confirmed a significant increase in porosity index of approximately 35% in both mutants (Figure 4b; Table 3). Such increased porosity has been closely associated with chalky endosperm formation and reduced mechanical integrity in rice grains [26]. These microstructural defects provide a direct link between molecular alterations in starch organization and the macroscopic phenotypes of reduced viscosity and opaque grain appearance observed in the mutants. Consistent with these structural and functional changes, *OsFLO2* disruption was accompanied by coordinated transcriptional reprogramming of starch biosynthetic genes. qRT-PCR analysis revealed reduced expression of *GBSSI*, *SSIIa*, *SSIIIa*, *BEI*, *BEIIb*, *ISA*, and *PUL*, alongside upregulation of *GBSSII*, *SSIVa*, and *BEIIa* across multiple developmental stages (Figure 5a). Multivariate analysis further demonstrated that these transcriptional shifts were closely associated with variation in RVA-derived pasting parameters (Figure 5b). Genes involved in amylose synthesis and intermediate-to-long chain elongation aligned with viscosity-related axes, whereas genes contributing to branching and trimming contributed to secondary variation. Together, these results indicate that *OsFLO2* influences starch properties through coordinated regulation of multiple biosynthetic components rather than through direct enzymatic activity.

Taken together, the integration of grain phenotypes (Figure 1 and Table 1), starch functional properties (Figure 2 and Table 2), amylopectin architecture (Figure 3), granule microstructure (Figure 4 and Table 3), and transcriptional regulation (Figure 5) provides a coherent picture of *OsFLO2* function in rice endosperm development. Rather than acting as a catalytic factor, *OsFLO2* appears to contribute to the coordination of starch biosynthesis and granule organization, thereby maintaining endosperm structural integrity and grain quality. This integrated view helps explain why diverse *flo2* alleles consistently produce floury endosperm phenotypes and advances our understanding of how non-catalytic regulatory proteins can exert broad effects on starch organization during seed development.

## 5. Conclusions

This study demonstrates that loss of *OsFLO2* function leads to reproducible alterations in rice endosperm structure and starch-related properties. Two independent CRISPR/Cas9-generated knockout lines exhibited increased grain chalkiness, reduced starch pasting viscosity, modified thermal behavior, and altered amylopectin chain-length distribution, accompanied by increased starch granule porosity. These changes were consistently observed across alleles and were associated with coordinated transcriptional shifts in starch biosynthetic genes. Collectively, the results indicate that *OsFLO2* contributes to rice endosperm quality by influencing starch organization and granule packing rather than by directly catalyzing starch synthesis. This work provides experimental evidence linking *OsFLO2* disruption to integrated changes in starch structure and grain quality traits.

## Figures and Tables

**Table 1:** Agronomic and grain quality traits of *OsFLO2* mutants and WT.

Trait	WT	*flo2-7*	*flo2-9*
Chalkiness (%)	4.8 ± 0.6	27.3 ± 1.8 ***	29.1 ± 2.0 ***
1000-grain weight (g)	23.5 ± 0.5	22.0 ± 0.4 *	21.9 ± 0.6 *
Filled grain ratio (%)	93.2 ± 1.1	90.5 ± 1.4	89.8 ± 1.7

Chalkiness (%), 1000-grain weight (g), and filled grain ratio (%) were measured in WT and two independent *OsFLO2* knockout lines (*flo2-7* and *flo2-9*). Three independent biological replicates were used. Values represent the mean ± SD based on three technical replicates. Significant differences, as determined by Student’s *t*-test, are indicated by asterisks (*, *p* < 0.05; ***, *p* < 0.001).

**Table 2:** RVA and DSC parameters of WT and *OsFLO2* mutant lines.

Parameter	WT	*flo2-7*	*flo2-9*
Peak viscosity (cP)	1193 ± 107	263 ± 95 ***	293 ± 62 ***
Final viscosity (cP)	1262 ± 140	385 ± 130 ***	415 ± 150 ***
Breakdown (cP)	617 ± 32	246 ± 13 ***	311 ± 22 ***
Pasting temperature (°C)	77.2 ± 0.8	68.9 ± 0.8 ***	70.5 ± 0.7 ***
△H (J/g)	188.2 ± 28	140.6 ± 21 **	158.1 ± 13 **

RVA parameters, including peak viscosity, final viscosity, breakdown, and pasting temperature, were obtained from RVA pasting profiles of WT and *OsFLO2* mutants. Gelatinization enthalpy (ΔH) was determined by DSC. Values represent the mean ± SD. Statistical significance was evaluated using Student’s *t*-test (**, *p* < 0.01; and ***, *p* < 0.001).

**Table 3:** Morphological characteristics of starch granules in WT and *OsFLO2* mutant lines.

Trait	WT	*flo2-7*	*flo2-9*
Mean diameter (μm)	6.5 ± 0.3	6.2 ± 0.2	6.1 ± 0.3
Porosity index	1.00 ± 0.05	1.36 ± 0.07 ***	1.33 ± 0.06 ***

Mean granule diameter and porosity index were quantified from SEM micrographs. Measurements were obtained from multiple fields of view per sample to capture representative granule populations. Values represent the mean ± SD from three biological replicates (n = 3). Statistical significance was determined using Student’s *t*-test (***, *p* < 0.001).

## Data Availability

The original contributions presented in the study are included in the article/Supplementary Materials; further inquiries can be directed to the corresponding authors.

## Supplementary Materials

The following supporting information can be downloaded at: https://www.mdpi.com/article/10.3390/genes17010063/s1. Supplementary Figure S1: Generation and selection of rice lines lacking the *OsFLO2* gene. (A) Schematic diagram of the CRISPR/Cas9 T-DNA construct used for genome editing. This cassette contains Cas9 driven by the CaMV 35S promoter and sgRNA driven by the OsU3 promoter. The *PPT* resistance gene (*Bar*) was included as a selectable marker. (B) Transformation of rice plants using *Agrobacterium* to introduce the pBOsC::sgRNA vector and produce tissue cultures. a-b, callus formation; c, selection of embryogenic callus and infection; d-e, co-culture after infection and shoot induction; f, regenerated plants in rooting medium; g-h, regenerated plants in rooting medium and acclimation in soil. (C) PCR screening of T_0_ rice lines to confirm transgene insertion using Nos-Bar-specific primers. M, molecular marker; PC, positive control; Supplementary Figure S2: Selection of *OsFLO2* knockout individuals and null lines. (A) Information on mutant sequences identified through NGS in the T_0_ generation. Two homologous individuals were identified in sg1. The lines were named and self-pollinated to advance the generation. (B) PCR analysis of the *Bar-Nos*-T gene region for the establishment of null segregants. DNA was extracted from leaves of T_1_ plants that were self-pollinated with T_0_ and amplified by PCR using primers specific for the *Bar-Nos*-T region. The absence of the corresponding PCR band indicates the isolation of plants lacking T-DNA (null). M, molecular marker; P.C, positive control; N.C, negative control; DW, indicator of contamination; Supplementary Table S1: Selected sgRNAs in this study; Supplementary Table S2: Regeneration ratios of mutant genotypes and mutant types at the target site in T_0_ mutant plants; Supplementary Table S3: Primer sequence used in this study.
