# Supplementary material for: FLOURY ENDOSPERM 2 Coordinates Starch Biosynthesis to Maintain Endosperm Structural Integrity in Rice
Source: Genes (Basel). 2026 Jan 5;17(1):63. doi: 10.3390/genes17010063 (PMC12840598; doi:10.3390/genes17010063)
Supplement: Supplementary file 1 [file genes-17-00063-s001.zip › genes-4056695-supplementary.pdf]

Supplementary Data

FLOURY ENDOSPERM 2 Coordinates Starch and Protein Biosynthetic Complexes to Maintain Endosperm Structural Integrity

Hye-Mi Lee [1], Jin-Young Kim [1], Hak-Dong Kim [1], Hak-Soo Kim [1], Jong-Geun Park [1], Yu-Jin Jung [1,2,*] and Kwon-Kyoo Kang [1,2,*]

Contents

Supplementary Figure 1: Generation and selection of rice lines lacking the *OsFLO2* gene. (A) Schematic diagram of the CRISPR/Cas9 T-DNA construct used for genome editing. This cassette contains Cas9 driven by the CaMV 35S promoter and sgRNA driven by the *Oryza sativa* U3 (OsU3) promoter. The *PPT* resistance gene (*Bar*) was included as a selectable marker. (B) Transformation of rice plants using *Agrobacterium* to introduce the pBOsC::sgRNA vector and produce tissue cultures. a-b, callus formation; c, selection of embryogenic callus and infection; d-e, co-culture after infection and shoot induction; f, regenerated plants in rooting medium; g-h, regenerated plants in rooting medium and acclimation in soil. (C) PCR screening of T_0 rice lines to confirm transgene insertion using Nos-Bar-specific primers. M; molecular marker; PC; positive control.

Supplementary Figure 2: Selection of *OsFLO2* knockout individuals and null lines. (A) Information on mutant sequences identified through NGS in the T_0 generation. Two homologous individuals were identified in sg1. The lines were named and self-pollinated to advance the generation. (B) PCR analysis of the *Bar-Nos*-T gene region for the establishment of Null segregants. DNA was extracted from leaves of T_1 plants that were self-pollinated with T_0 and amplified by PCR using primers specific for the *Bar–Nos*–T region. The absence of the corresponding PCR band indicates the isolation of plants lacking T-DNA (null). M, molecular marker; P.C, positive control; N.C, negative control; DW, indicator of contamination.; Supplementary Table 1: Selected sgRNAs in this study

Supplementary Table 1: Selected sgRNAs in this study.

Supplementary Table 2: Regeneration ratios of mutant genotypes and mutant types at the target site in T0 mutant plants.

Supplementary Table 3: Primer sequence used in this study.

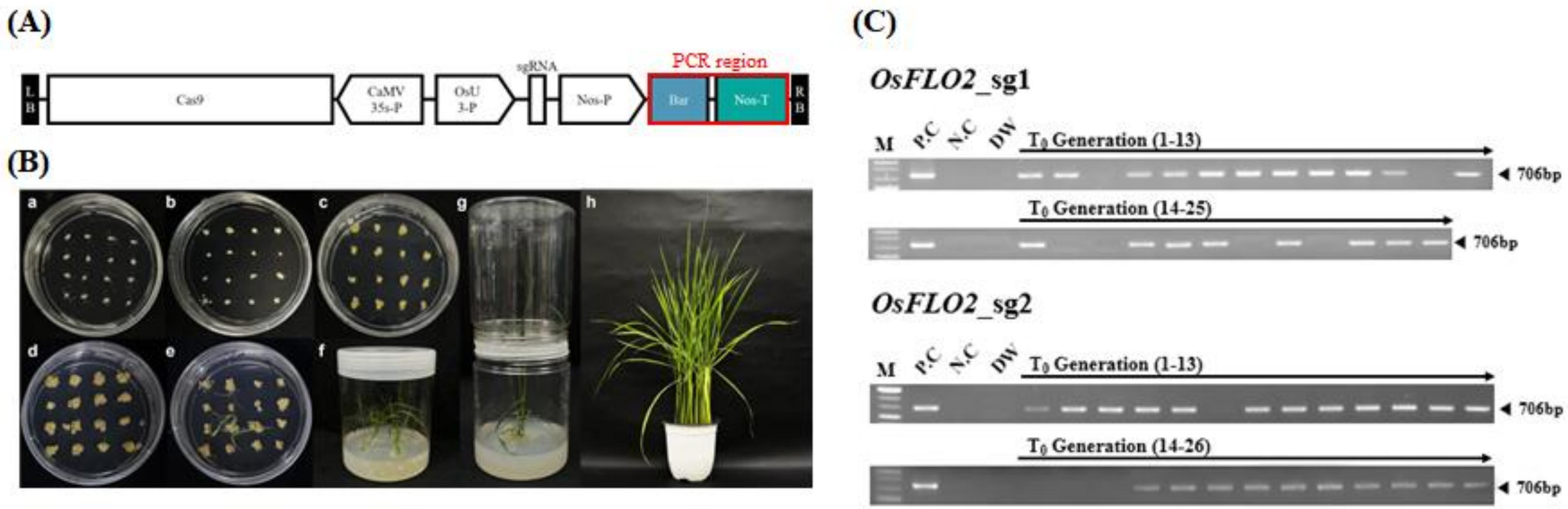

Supplementary Figure 1: Generation and selection of rice lines lacking the *OsFLO2* gene. (A) Schematic diagram of the CRISPR/Cas9 T-DNA construct used for genome editing. This cassette contains Cas9 driven by the CaMV 35S promoter and sgRNA driven by the *Oryza sativa* U3 (OsU3) promoter. The *PPT* resistance gene (*Bar*) was included as a selectable marker. (B) Transformation of rice plants using *Agrobacterium* to introduce the pBOsC::sgRNA vector and produce tissue cultures. a-b, callus formation; c, selection of embryogenic callus and infection; d-e, co-culture after infection and shoot induction; f, regenerated plants in rooting medium; g-h, regenerated plants in rooting medium and acclimation in soil. (C) PCR screening of T_0 rice lines to confirm transgene insertion using Nos-Bar-specific primers. M; molecular marker; PC; positive control.

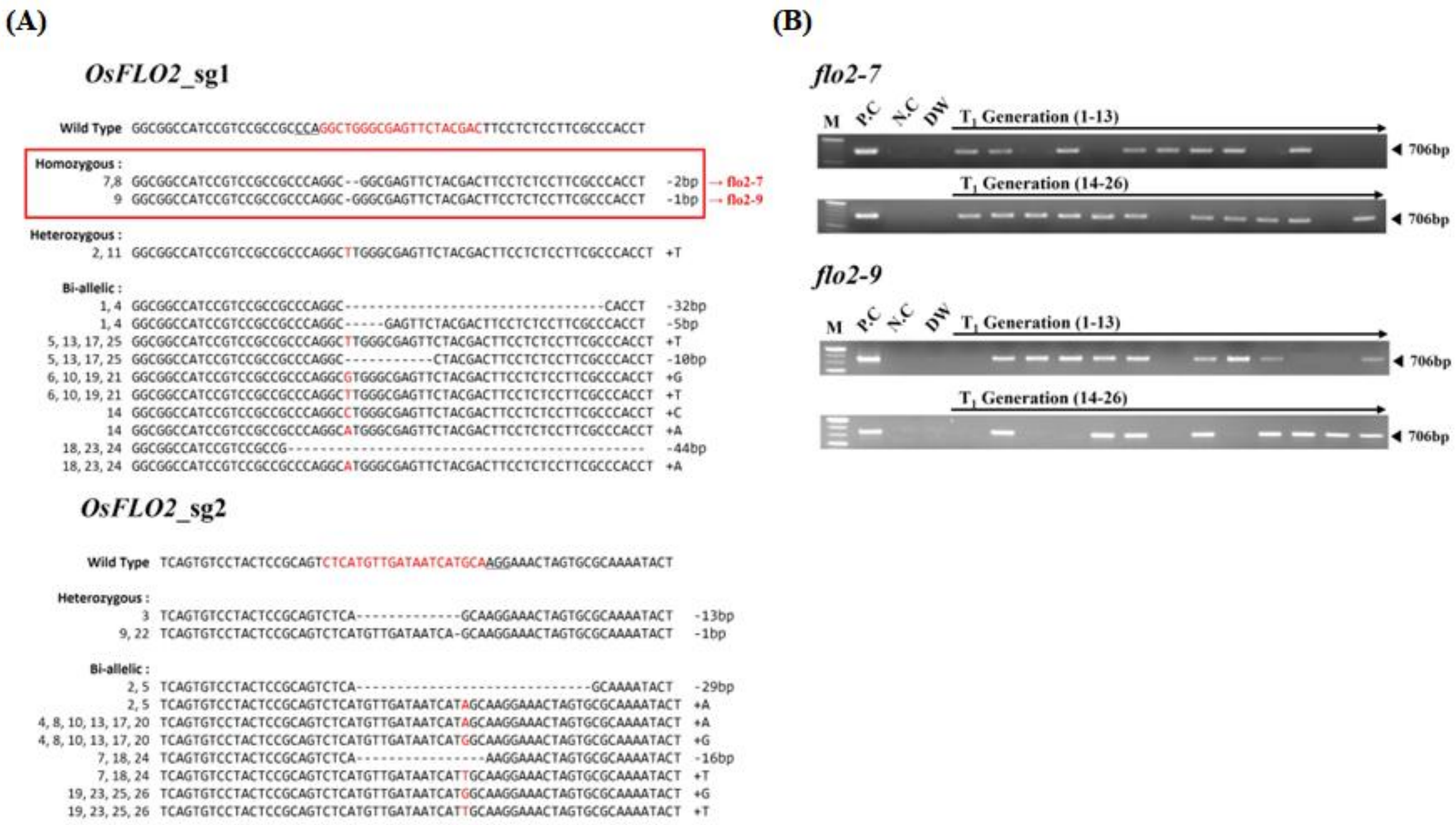

Supplementary Figure 2: Selection of *OsFLO2* knockout individuals and null lines. (A) Information on mutant sequences identified through NGS in the T_0 generation. Two homologous individuals were identified in sg1. The lines were named and self-pollinated to advance the generation. (B) PCR analysis of the *Bar-Nos*-T gene region for the establishment of Null segregants. DNA was extracted from leaves of T_1 plants that were self-pollinated with T_0 and amplified by PCR using primers specific for the *Bar–Nos*–T region. The absence of the corresponding PCR band indicates the isolation of plants lacking T-DNA (null). M, molecular marker; P.C, positive control; N.C, negative control; DW, indicator of contamination.; Supplementary Table 1: Selected sgRNAs in this study

Supplementary Table 1: Selected sgRNAs in this study.

Gene	Target sgRNA (5' to 3')	Direction	GC Contents (%, w/o PAM)	Out Of Frame Score	Mismatches			
					**0**	**1**	**2**	**3**
*OsFLO2*_sg1	CCAGGCTGGGCGAGTTCTACGAC	-	65	85.3	1	0	0	0
*OsFLO2*_sg2	CTCATGTTGATAATCATGCAAGG	+	35	83.7	1	0	0	0

Supplementary Table 2: Regeneration ratios of mutant genotypes and mutant types at the target site in T0 mutant plants.

Gene	sgRNA No.	No. of infected Calli	No. of regenerated T_0 plants	No of Transgenic plants	No. of Editing plants (%)	Mutant genotype ratios (%)		
						Homo	Bi-allelic	Hetero
OsFLO2	sgRNA1	80	30	25	19 (76.0)	3 (15.8)	14 (73.7)	2 (10.5)
	sgRNA2	80	33	26	19 (73.1)	1 (5.3)	15 (78.9)	3 (15.8)
	Total	160	63 (39.4)	51 (83.6)	38 (84.6)	4 (10.5)	29 (76.3)	5 (13.2)

Supplementary Table 3: Primer sequence used in this study.

Primer name	Sequence (5'-3') Foward	Reverse	Product size (bp)	Purpose
pBOsC sgSEQ - RGEN scaaffold regoin	CAGCTTGGCTCTAGTCGACC	CGGTGCCACTTTTTCAAGTT	510	Vector construction
OsFLO2 sg1	ggcagGGCTGGGCGAGTTCTACGACc	aaacGTCGTAGAACTCGCCCAGCC	29	
OsFLO2 sg2	ggcagCTCATGTTGATAATCATGCAc	aaacTGCATGATTATCAACATGAG	29	
T-DNA confirm-*Nos-Bar*	TTGCGCGCTATATTTTGTTTT	CGTCAACCACTACATCGAGA	706	T-DNA confirm
OsFLO2 sg1 1st	TTGGGCCTGAATTTTGGTAG	AAACTTTCACCTGGCCACAC	624	
OsFLO2 sg2 1st	TGGCAAACAAACCAGTGATATCT	AGCACCTTCAACATGGCATT	600	
OsFLO2 sg1 2nd	acactctttccctacacgacgctcttccgatctAGCACAAGTCGTCCAAGCAC	gtgactggagttcagacgtgtgctcttccgatctCTCGCAAAAAGGGGGAAAC	312	
OsFLO2 sg2 2nd	acactctttccctacacgacgctcttccgatctCATCGCTAGTACTCAGTGTCCT	gtgactggagttcagacgtgtgctcttccgatctTTCTTGTCCTCAGATGCCGG	268	
OsACTIN qRT-PCR	CAACACCCCTGCTATGTACG	ATCACCAGAGTCCAACACAA	191	qRT-PCR analysis
OsFLO2 qRT-PCR	GTCGCACCCTTACAGTACAG	GTACAATGTGGACCGAGGAC	187	
OsGBSSI qRT-PCR	GGACCTGACACTGGAGTTG	TTTGAAGTATGGGTTGTTGTTGAG	184	
OsGBSSII qRT-PCR	GCTTGCTGGCTTGTTTAG	TGAAGTAGACGAGAGAAATGG	180	
OsBEI qRT-PCR	CCTGCTTCACCTACCATCAA	CGACAAGGCTCCACTGAC	190	
OsBEIIa qRT-PCR	GCCAATGCCAGGAAGATGA	GCGCAACATAGGATGGGTTT	194	
OsBEIIb qRT-PCR	ATGCTAGAGTTTGACCGC	AGTGTGATGGATCCTGCC	200	
OsSSIIa qRT-PCR	CCTATTCCTGCGGTAGAAGA	CCGAATCGTCATCCTGGT	188	
OsSSIIIa qRT-PCR	GAGGAAGTGGATGTGGTAGATGAA	TCTGTTTCGGTGATTGAAGCATTT	183	
OsSSIVa qRT-PCR	GGGAGCGGCTCAAACATAAA	CCGTGCACTGACTGCAAAAT	197	
OsISA1 qRT-PCR	TGCTCAGCTACTCCTCCATCATC	AGGACCGCACAACTTCAACATA	182	
OsPUL qRT-PCR	ACCTTTCTTCCATGCTGG	CAAAGGTCTGAAAGATGGG	192	
